# Eosinophil-expressed galectin-3 regulates cell trafficking and migration

**DOI:** 10.3389/fphar.2013.00037

**Published:** 2013-04-05

**Authors:** Xiao Na Ge, Sung Gil Ha, Fu-Tong Liu, Savita P. Rao, P. Sriramarao

**Affiliations:** ^1^Department of Veterinary and Biomedical Sciences, University of MinnesotaSt. Paul, MN, USA; ^2^Department of Dermatology, University of California DavisSacramento, CA, USA; ^3^Department of Medicine, University of MinnesotaSt. Paul, MN, USA

**Keywords:** eosinophils, galectin-3, allergic airway inflammation, cell trafficking, migration

## Abstract

Galectin-3 (Gal-3), a β galactoside-binding lectin, is implicated in the pathogenesis of allergic airway inflammation and allergen-challenged mice deficient in Gal-3 (Gal-3^-/-^) exhibit decreased airway recruitment of eosinophils (Eos). Gal-3 is expressed and secreted by several cell types and can thus function extracellularly and intracellularly to regulate a variety of cellular responses. We sought to determine the role of Eos-expressed Gal-3 in promoting Eos trafficking and migration in the context of allergic airway inflammation using bone marrow (BM)-derived Eos from wild-type (WT) and Gal-3^-/-^ mice. Airway recruitment of Eos in acute (4 weeks) and chronic (8–12 weeks) allergen-challenged WT mice correlated with Gal-3 expression in the lungs. BM-derived Eos were found to express Gal-3 on the cell surface and secrete soluble Gal-3 when exposed to eotaxin-1. Compared to WT Eos, Gal-3^-/-^ Eos exhibited significantly reduced rolling on vascular cell adhesion molecule 1 (VCAM-1) and decreased stable adhesion on intercellular adhesion molecule 1 (ICAM-1) under conditions of flow *in vitro*. Evaluation of cytoskeletal rearrangement demonstrated that relatively fewer adherent Gal-3^-/-^ Eos undergo cell spreading and formation of membrane protrusions. In addition, cell surface expression of integrin receptor αM (CD11b) was lower in Gal-3^-/-^ Eos, which is likely to account for their altered adhesive interactions with VCAM-1 and ICAM-1. Gal-3^-/-^ Eos also exhibited significantly decreased migration toward eotaxin-1 compared to WT Eos irrespective of similar levels of CCR3 expression. Further, eotaxin-induced migration of WT Eos remained unaffected in the presence of lactose, suggesting a role for intracellular Gal-3 in regulating Eos migration. Overall, our findings indicate that Gal-3 expression in the lungs correlates with Eos mobilization during allergic airway inflammation and signaling involving intracellular Gal-3 and/or secreted Gal-3 bound to the cell surface of Eos appears to be essential for Eos trafficking under flow as well as for migration.

## INTRODUCTION

Galectins (1–15) are members of a highly conserved family of animal lectins defined by their affinity for β-galactose-containing oligosaccharides (Liu et al., 2012a). They do not contain a classical signal sequence nor a transmembrane domain and are located intracellularly (cytoplasm and nucleus), but are also present extracellularly (Ochieng et al., 1993; Hughes, 1999; Leffler, 2001). Because of this, galectins can function extracellularly and intracellularly and thus participate in a variety of pathways regulating cellular responses such as cell adhesion, migration, proliferation, differentiation, and apoptosis, and play an important role in acute and chronic inflammation (Liu, 2000; Liu et al., 2012a). Among galectins, galectin-3 (Gal-3) is unique since it is a chimeric galectin containing one carbohydrate-recognition domain (CRD) linked to a proline, glycine, and tyrosine rich N-terminal region.

Previous studies have implicated a role for endogenous Gal-3 in the pathogenesis of allergic airway inflammation. *In vivo* studies have demonstrated that Gal-3 expression in the lungs is up-regulated during allergic asthma and Gal-3 deficient (Gal-3^-/-^) mice exhibit significantly reduced pulmonary eosinophilia and airway hyperresponsiveness (AHR) in response to acute allergen [ovalbumin (OVA)] challenge relative to wild-type (WT) mice (Zuberi et al., 2004). In addition, we have previously shown that Gal-3^-/-^ mice exposed to chronic allergen (OVA) challenge have attenuated airway eosinophilia and exhibit less severe remodeling of the airways, i.e., reduced mucus secretion, sub-epithelial fibrosis, smooth muscle thickness, and peribronchial angiogenesis, compared to WT counterparts (Ge et al., 2010). At a cellular level, studies from our laboratory have demonstrated that Gal-3 is present on the cell surface of human eosinophils (Eos) from allergic donors at higher levels than Eos from normal subjects and functions as a cell surface adhesion molecule to support Eos rolling and adhesion under conditions of flow (Rao et al., 2007). Overall, these studies suggest that Gal-3 plays a pro-inflammatory role during allergic asthma and chronic allergic airway inflammation.

The recruitment of Eos to inflamed tissues involves specific and sequential adhesive interactions between cell surface adhesion receptors and vascular counter ligands in inflamed blood vessels followed by their sequestration to extravascular sites (Broide and Sriramarao, 2001, 2008; Rosenberg et al., 2007). The ligation of integrin receptor α4β1 with vascular cell adhesion molecule 1 (VCAM-1) mediates Eos rolling, adhesion, and migration, contributing to selective recruitment of Eos (Walsh et al., 1991; Sriramarao et al., 1994; Yamamoto et al., 2005). In addition, a role for β1 and β2 integrins in mediating Eos trafficking has been identified (Barthel et al., 2008). We and others have shown that, extracellularly or exogenously added Gal-3 can bind to integrin and other glycoprotein receptors on the cell surface of human Eos to facilitate cell trafficking and activation, respectively (Rao et al., 2007; Yoon et al., 2007). Since Gal-3 is secreted by multiple cell types (macrophages, activated T cells), *in vivo*, this secreted Gal-3 can bind to glycan ligands on Eos and exert its effects. In the current study, we have evaluated the specific role of Eos-expressed Gal-3, whether intracellular or extracellular, in mediating Eos trafficking and migration, especially in the context of the reduced airway eosinophilia observed in Gal-3^-/-^deficient mouse models of allergic airway inflammation.

## MATERIALS AND METHODS

### MOUSE MODEL OF ALLERGIC AIRWAY INFLAMMATION

WT C57BL/6 mice (8–12 weeks) were sensitized and challenged with OVA (Grade V, Sigma Chemical Co., St Louis, MO, USA) up to 4 weeks as descried previously (Bahaie et al., 2012; acute model). For chronic allergen exposure, acute allergen-challenged mice continued to receive biweekly challenges for an additional 4 weeks (8 weeks total allergen exposure; Cho et al., 2010) or 8 weeks (12 weeks total allergen exposure; Ge et al., 2010; chronic models). Control mice were sensitized and challenged with phosphate-buffered saline (PBS) instead of OVA. All studies involving mice were performed following standards and procedures approved by the Institutional Animal Care and Use Committee at the University of Minnesota.

### SAMPLE COLLECTION

Mice were sacrificed 24 h after the last allergen challenge. Eos counts in the broncho alveolar lavage fluid (BALF) were determined based on morphologic and histologic criteria after staining with Hema 3 System (Thermo Fisher Scientific Co., Pittsburgh, PA, USA). BALF supernatants were stored at -70°C and lung tissue was snap-frozen till further evaluation.

### MOUSE BONE MARROW Eos

Eos were cultured from bone marrow (BM) of WT C57BL/6 and Gal-3^-/-^ mice (Ge et al., 2010) as previously described (Dyer et al., 2008). Cells between day 12 and 15 of culture differentiated based on Hema 3 staining and evaluated for expression of both Eos-expressed major basic protein (MBP) and Siglec-F (Bahaie et al., 2012) were used in studies.

### ENZYME-LINKED IMMUNOSORBENT ASSAY

Gal-3 levels in BALF from control and allergen-challenged mice were evaluated by enzyme-linked immunosorbent assay (ELISA) using affinity-purified goat anti-Gal-3 antibody as the capture antibody and affinity-purified rabbit anti-Gal-3 antibody as the primary detection antibody as described previously (Zuberi et al., 2004).

### WESTERN BLOT ANALYSIS

Lung tissue and BM-derived Eos lysates were prepared in radioimmunoprecipitation assay (RIPA) buffer and total protein in the supernatants was measured (BCA Protein Assay Kit, Pierce, Rockford, IL, USA). Lung tissue and Eos lysates (20 μg/per lane) as well as BALF from allergen-challenged mice and Eos culture supernatants (20 μl/lane) were electrophoresed on 12% Tris-Glycine gels under reduced conditions. Western blot analysis was carried out with polyclonal antibodies against Gal-3 (1 μg/ml; Liu et al., 1995) followed by goat anti-rabbit IRDye 800CW (1:8000, LI-COR Biosciences, Lincoln, NE, USA). For lung tissue and cell lysates, expression of β-actin was monitored as an internal control using anti-mouse β-actin (0.05 μg/ml, BD Transduction Laboratories^TM^, San Diego, CA, USA) followed by goat anti-mouse IRDye-680 (1:8000, LI-COR Biosciences). Detection was carried out with an Odyssey Infrared Imaging System (LI-COR Biosciences). Densitometry of scanned images was performed using ImageJ and density of the Gal-3 bands in lung tissue was normalized against β-actin after background subtraction.

### CONFOCAL MICROSCOPY

Bone marrow-derived Eos were cytocentrifuged on to glass slides, fixed with 4% paraformaldehyde in PBS for 20 min at room temperature and blocked with 1.5% goat serum in PBS. Cells were then incubated overnight at 4°C with mAb against Gal-3 (10 μg/ml, Clone B2C10; Hsu et al., 2000). Bound antibodies were detected using FITC-conjugated goat anti-mouse IgG (Jackson ImmunoResearch Laboratories, Inc., Westgrove, PA, USA). Cells were stained with 4′,6-diamidino-2-phenylindole (DAPI) to visualize nuclei and examined by confocal microscopy [FLUOVIEW FV1000/BX61 – Confocal Laser Scanning Biological Microscope equipped with an UPlanSApo lens (20×/0.85 [oil]) and a PlanApo N lens (60×/1.42 [oil]), Olympus, Melville, NY, USA] at ambient temperature. FV10-ASW 2.0 software was used for image acquisition Olympus.

### FLOW CYTOMETRY

To examine surface expression of Gal-3 by BM-derived Eos, non-permeabilized cells were suspended in cold PBS without Ca^2+^ and Mg^2+^ and incubated with polyclonal antibodies against Gal-3 (10 μg/ml) followed by PE-conjugated goat anti-rabbit IgG (5 μg/ml, Jackson ImmunoResearch Laboratories, Inc.,) with rabbit IgG as the negative control. To examine cell surface receptor expression, Eos from WT and Gal-3^-/-^ mice were incubated with mAbs against CD49d (α4, 10 μg/ml, Clone PS/2; Sriramarao et al., 1994), CD11a (αL, 10 μg/ml, eBioscience, San Diego, CA, USA), CD11b (αM, 10 μg/ml, eBioscience), or L-selectin (10 μg/ml, clone MEL-14; Sriramarao et al., 1994), respectively, followed by FITC-conjugated goat anti-rat IgG (Jackson ImmunoResearch Laboratories, Inc.). Rat IgG2b (eBioscience, for α4 and CD11b) and rat IgG2a (eBioscience, for CD11a and L-selectin) were used as isotype-matched controls. For CCR3 expression, Eos were incubated with FITC-conjugated rat anti-mouse CCR3 (2.5 μg/ml, R&D Systems, Minneapolis, MN, USA) with FITC-conjugated rat IgG2a (eBioscience) as the isotype-matched control. Cells were examined using a FACScan flow cytometer (BD Biosciences) and FlowJo software (version 8.8.2, Tree Star, Ashland, OR, USA).

### FLOW CHAMBER ASSAY

Rolling of BM-derived Eos from WT and Gal-3^-/-^ mice on recombinant mouse (rm) VCAM-1 and intercellular adhesion molecule 1 (ICAM-1) under conditions of flow (wall shear stress ~2.0 dynes/cm^2^; Sriramarao et al., 1996) was evaluated in an *in vitro* parallel plate flow chamber as described previously (Rao et al., 2007; Bahaie et al., 2011). The interaction of Eos with VCAM-1- and ICAM-1-coated coverslips was observed using a Leitz Wetzlar inverted microscope and images were recorded for subsequent offline analysis to manually determine the number of interacting cells. Results were expressed as the number of rolling or adherent cells/2 min.

### STATIC ADHESION AND CELL MORPHOLOGY

Bone marrow-derived Eos (1 × 10^5^ cells) from WT or Gal-3^-/-^ mice were added to rm VCAM-1-coated coverslips (10 μg/ml in PBS, 100 μl per coverslip) and allowed to adhere for 30 min at 37°C. Coverslips were washed and adherent cells were fixed with 4% paraformaldehyde in PBS for 20 min. Adherent cells were stained with Alexa Fluor 488 phalloidin as well as DAPI to visualize nuclei and examined by confocal microscopy as described (Kang et al., 2012). To assess differences in cell morphology between WT and Gal-3^-/-^ Eos, adherent cells in five randomly selected fields of each coverslip were counted and the number of cells that exhibited spreading with several membrane protrusions from a round cell body was identified and expressed as a percentage of the total number of adhered cells in the field. Results are expressed as percent adhered cells exhibiting change in morphology relative to WT Eos.

### *IN VITRO* MIGRATION ASSAY

Migration of WT BM-derived Eos in response to eotaxin-1 (100 nM, PeproTech) was evaluated using 96-well Transwell^®^ Chambers as described previously (Bahaie et al., 2011). In some experiments, lactose (or maltose as control) at a final concentration of 3 mM was added to the cell suspension to inhibit binding of secreted/extracellular Gal-3 (Rao et al., 2007; Bahaie et al., 2011) before placing cells in the chamber. The number of migrated cells in each case was evaluated after 3–4 h using an Olympus CK2 inverted microscope under a magnification of 400×. Cells in a fixed number of randomly selected non-overlapping fields were counted for each well for each experiment. The assay was performed three times in duplicate. Results are expressed as percent cell migration relative to WT Eos or as the average number of cells/field.

### STATISTICAL ANALYSIS

Results are expressed as mean ± SEM. significance was determined using the unpaired Student’s *t*-test. A *p* value <0.05 was considered as significant.

## RESULTS

### AIRWAY EOSINOPHILIA IN ALLERGEN-CHALLENGED MICE IS ASSOCIATED WITH ELEVATED Gal-3 EXPRESSION IN THE LUNGS

**FIGURE 1:**
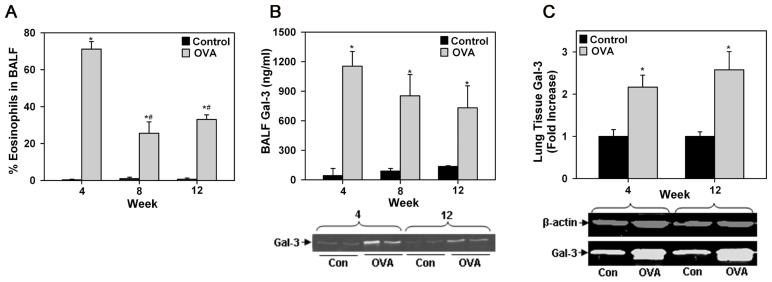
**Airway eosinophilia in allergen-challenged mice is associated with elevated Gal-3 expression.** **(A)** Eos in BALF of WT C57BL/6 mice after allergen challenge for 4, 8, or 12 weeks (*n* = 5 mice/group). **(B)** Gal-3 levels in BALF of allergen-challenged and control mice by ELISA (top, *n* = 6 mice/group) as well as Western blot analysis (bottom). Representative results for two mice from 4 and 12 week allergen-challenged groups along with corresponding controls (*n* = 3–4 mice/group) are shown. **(C)** Quantitation of Gal-3 expression in lung tissue of allergen-challenged and control mice by densitometric analysis of Western blots. Representative results for each group are shown below (*n* = 3–4 mice/group). Data represent mean ± SEM. *p <0.01 in **(A)** and **(C)** and <0.03 in **(B)** versus control mice; ^#^*p* <0.01 versus 4 week allergen-challenged mice in **(A)**.

Differential cell counts in BALF of acute or chronic allergen-exposed mice indicated increased recruitment of Eos compared with control mice. Although, the number of Eos in the lungs of chronic allergen-challenged mice was lower than in acute allergen-challenged mice, it was still significantly higher compared to corresponding control mice (**Figure 1A**). Given that mice deficient in Gal-3 have decreased eosinophilia (Zuberi et al., 2004; Ge et al., 2010), we first evaluated Gal-3 expression in the lungs of allergen-challenged mice. As previously reported in the case of acute allergic airway inflammation (Zuberi et al., 2004), even chronic allergen-challenged mice had elevated levels of soluble Gal-3 in the BALF compared to corresponding control mice as indicated by ELISA (**Figure 1B**, top) and Western blot analysis (**Figure 1B**, bottom). Although, BALF Gal-3 levels in allergen-challenged mice correlated with airway eosinophilia, being somewhat higher in acute than in chronic allergen-challenged mice, a statistically significant difference between the allergen-challenged groups was not noted. In addition, Gal-3 expression in the lung tissue of acute and chronic allergen-challenged mice determined by Western blot analysis was comparable and significantly higher than in corresponding control mice (**Figure 1C**). These findings, together with previous studies demonstrating decreased eosinophilia in allergen-challenged Gal-3^-/-^ mice (Zuberi et al., 2004; Ge et al., 2010) suggest that endogenous Gal-3, whether extracellular (as soluble protein in BALF) or intracellular (contained within or bound to surface glycan ligands of certain cell types in lung tissue), is critically involved in Eos recruitment to the airways of acute and chronic allergen-challenged mice.

### MOUSE Eos EXPRESS Gal-3

**FIGURE 2:**
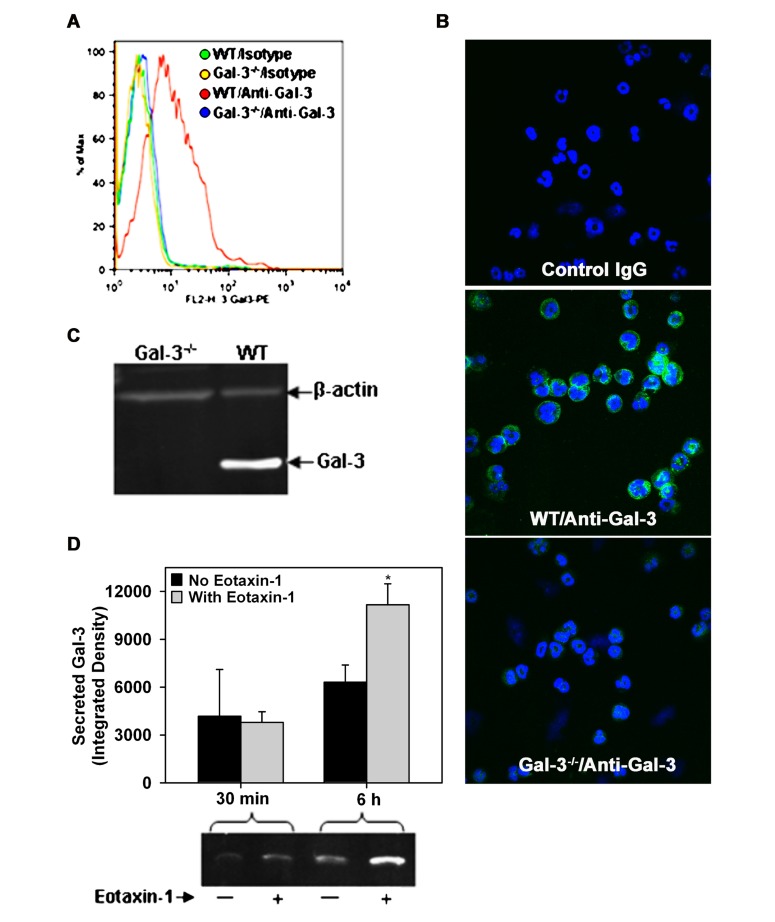
**Expression and release of Gal-3 by BM-derived mouse Eos**. **(A)** Cell surface expression of Gal-3 in BM-derived Eos from WT and Gal-3^-/-^ mice by flow cytometry with rabbit polyclonal antibody against Gal-3 and rabbit IgG as negative control. **(B)** Cell surface expression of Gal-3 in non-permeabilized BM-derived Eos by confocal microscopy using monoclonal antibodies against Gal-3. Representative images for isotype control (mouse IgG, top), WT Eos (middle), and Gal-3^-/-^ Eos (bottom) are shown at a magnification of 600×. **(C)** Gal-3 expression in WT and Gal-3^-/-^ Eos lysates by Western blot analysis using rabbit polyclonal antibodies against Gal-3. **(D)** Gal-3 in culture supernatant of Eos incubated with eotaxin-1 (100 nM) or media alone for 30 min or 6 h by Western blot analysis followed by densitometry (Mean ± SEM). *p <0.05 versus Eos cultured without eotaxin-1 in **(D)**. Representative data of two to four independent experiments in **(A–C)** and of three independent experiments in bottom panel of **(D)** performed with BM-derived Eos from different mice is shown.

While Gal-3 is known to be expressed on the cell surface of human Eos (Rao et al., 2007) and BALF Eos in allergen-challenged mice (Ge et al., 2010), thus far there are no studies demonstrating expression of Gal-3 by BM-derived mouse Eos. In order to further investigate the potential role of endogenous Eos-expressed Gal-3 in mediating Eos trafficking and recruitment, we first established that mouse BM-derived Eos from naïve mice (non-allergen-challenged) express Gal-3. Non-permeabilized BM-derived Eos from WT mice were found to be positive for Gal-3 expression when stained with polyclonal antibodies against Gal-3 and examined by flow cytometry relative to Eos from Gal-3^-/-^ mice stained with the same antibody or Eos stained with normal IgG which served as negative controls (**Figure 2A**). These studies were confirmed by confocal microscopy which demonstrated the presence of Gal-3 on the cell surface of non-permeabilized WT BM-derived Eos, but not Gal-3^-/-^ Eos (**Figure 2B**). Further, WT BM-derived Eos lysates analyzed for Gal-3 expression by Western blot analysis displayed a band of 29 kDa corresponding to the reported molecular mass for Gal-3 (Hsu et al., 2000; **Figure 2C**).

Since allergic airway inflammation is associated with elevated levels of soluble Gal-3 levels in the BALF (**Figure 1B**), we investigated whether activated Eos are a source for Gal-3. WT BM-derived Eos were cultured in medium alone (control) or medium containing eotaxin-1 for different time intervals and cell culture supernatants were analyzed for Gal-3 by Western blot analysis. After 30 min, Gal-3 levels were almost similar in the culture supernatant of eotaxin-1-treated and untreated Eos; however, after 6 h, there was a significant increase in secreted Gal-3 levels in the culture supernatant of eotaxin-1-treated Eos relative to corresponding control supernatant (**Figure 2D**, *p* <0.05). These findings confirm that BM-Eos express Gal-3 intracellularly which is secreted when cells are activated.

### Eos-EXPRESSED Gal-3 IS REQUIRED FOR EFFICIENT Eos ROLLING AND ADHESION UNDER CONDITIONS OF FLOW

**FIGURE 3:**
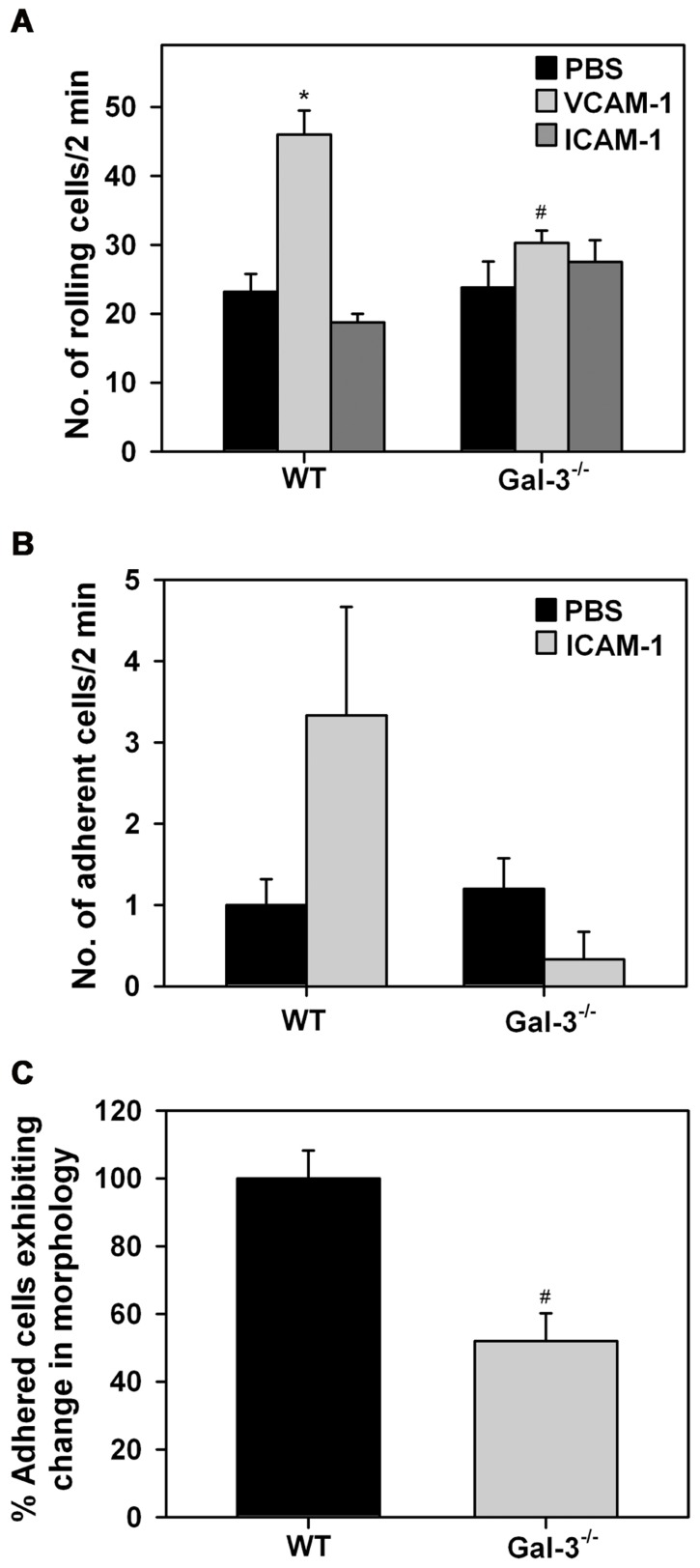
**Gal-3 deficient Eos exhibit decreased trafficking and altered cell morphology after adhesion**. **(A)** Rolling and **(B)** adhesion of BM-derived Eos from WT and Gal-3^-/-^ mice on rmVCAM-1- and rmICAM-1-coated coverslips under conditions of flow (~2.0 dynes/cm^2^) in an *in vitro* flow chamber. **(C)** BM-derived Eos from WT and Gal-3^-/-^ mice adherent on rmVCAM-1-coated coverslips were stained with Alexa Fluor 488 phalloidin and DAPI and examined by confocal microscopy. Adherent cells in five randomly selected fields of each coverslip were counted and the number of cells that exhibited spreading and membrane protrusions from a round cell body was identified and expressed as a percentage of the total number of adhered cells in that field. Results presented are relative to WT Eos. Combined data (mean ± SEM) of two to three independent experiments is shown in **(A–C)**. *p <0.05 versus PBS-coated coverslips; ^#^*p* <0.05 versus WT Eos.****

Leukocyte–endothelial interactions play a crucial role in cellular recruitment to sites of inflammation. Our previous studies have identified a role for cell surface-expressed Gal-3 in supporting human Eos rolling and adhesion via interaction with vascular endothelial adhesion molecule VCAM-1 under conditions of flow (Rao et al., 2007). Since Gal-3 secreted by other cells (in addition to Eos) can also bind to glycan ligands on Eos and mediate these effects, these studies do not reveal whether Eos-expressed Gal-3 participates in Eos trafficking. We examined rolling of WT and Gal-3^-/-^ BM-derived Eos on immobilized rm VCAM-1 or rm ICAM-1 under conditions of flow (**Figure 3A**). WT Eos exhibited a two-fold increase in rolling (*p* <0.01) on coverslips coated with rmVCAM-1 compared to background rolling on PBS-coated coverslips. In contrast, rolling of Gal-3^-/-^ Eos on VCAM-1 was only marginally higher than background rolling on PBS-coated coverslips and was significantly lower compared to WT Eos (*p* <0.01; **Figure 3A**). Rolling of both WT and Gal-3^-/-^ Eos on rmICAM-1, a molecule that does not support rolling, was similar to that observed on PBS. With respect to stable adhesion under conditions of flow, Gal-3^-/-^ Eos tended to adhere less effectively on ICAM-1 than WT Eos, although the difference was not statistically significant (**Figure 3B**). These data suggest that Eos-expressed Gal-3 is required for efficient Eos rolling on VCAM-1 and probably adhesion to ICAM-1 under conditions of flow.

Since Gal-3^-/-^ Eos exhibit decreased adhesive interactions with vascular endothelial adhesion molecules, we investigated whether Gal-3 plays a role in regulating cell morphology when Eos are allowed to interact with VCAM-1 under static conditions. WT and Gal-3^-/-^ Eos adherent on rmVCAM-1-coated coverslips were examined by confocal microscopy after phalloidin staining. A larger number of adherent WT Eos exhibited cell spreading with several membrane protrusions compared to adherent Gal-3^-/-^ Eos. In contrast, several Gal-3^-/-^ Eos retained a round cell body with limited membrane protrusions and spreading. Quantitation of these differences in cell morphology of adherent WT and Gal-3^-/-^ Eos revealed that, relative to WT Eos, a smaller percentage of Gal-3^-/-^ Eos exhibit changes in cell morphology upon adhesion to VCAM-1 (**Figure 3C**), suggesting that Gal-3 is required for activation-induced morphological changes that are essential for directed movement of cells.

### Gal-3 DEFICIENCY RESULTS IN DECREASED CELL SURFACE EXPRESSION OF αM BY Eos

**FIGURE 4:**
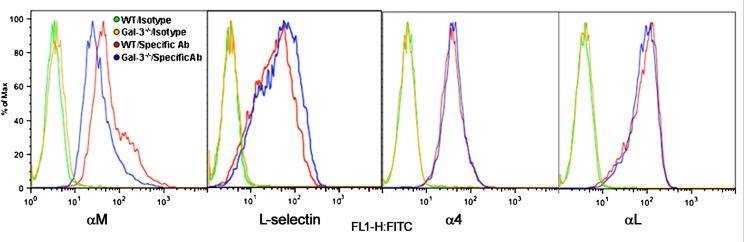
**Gal-3 deficient Eos express decreased levels of αM**. Expression of CD11b (αM), CD11a (αL), L-selectin, and CD49d (α4) on the cell surface of WT and Gal-3^-/-^ Eos was examined by flow cytometry using rat mAbs against CD11b, CD11a, CD49d, and L-selectin, respectively, followed by FITC-conjugated goat anti-rat IgG. Rat IgG2b (for CD11b and CD49d) and rat IgG2a (for L-selectin and CD11a) were used as isotype-matched control antibodies. Representative data of two to five independent experiments with Eos from different mice is shown.

Eos rolling and adhesion are mediated by multiple cell surface adhesion molecules. While our studies demonstrate a direct requirement for Eos-expressed Gal-3 in mediating Eos rolling and adhesion, we wanted to determine whether Gal-3 regulates the expression of cell surface adhesion molecules that promote Eos rolling and adhesion such as α4, L-selectin, αL, and αM (**Figure 4**). There was no difference in expression levels of L-selectin, α4, and αL between WT and Gal-3^-/-^ Eos. However, expression of αM by Gal-3^-/-^ Eos was considerably lower than by WT Eos.

### Eos-EXPRESSED Gal-3 IS REQUIRED FOR Eos MIGRATION

**FIGURE 5:**
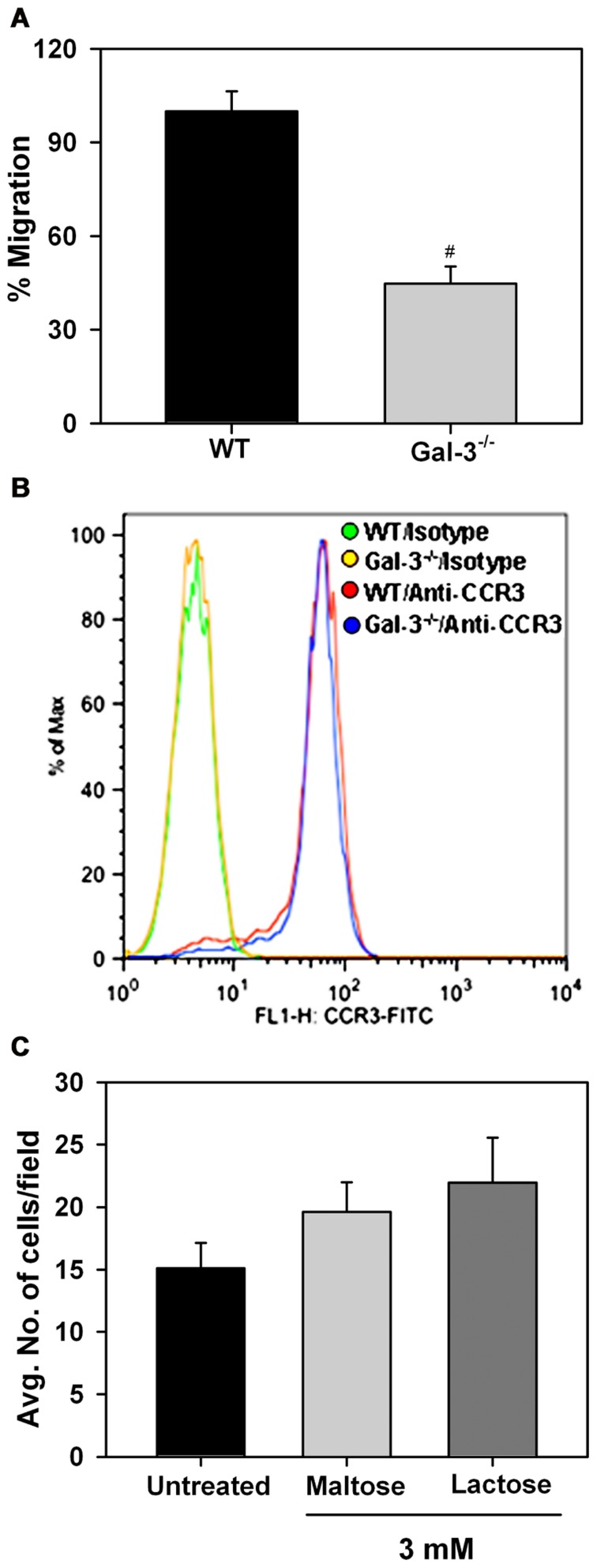
**Gal-3 deficient Eos exhibit decreased migration toward eotaxin-1**. **(A) **Migration of WT and Gal-3^-/-^ BM-derived Eos toward murine eotaxin-1 (100 nM) *in vitro* using 96-well Transwell ^®^ Chambers. Results are represented as percent cell migration relative to WT Eos. **(B)** Expression of CCR3 by WT and Gal-3^-/-^ Eos by flow cytometry using FITC-conjugated rat anti-mouse CCR3 with FITC-conjugated rat IgG2a as isotype control. Representative data of two independent experiments with Eos from different mice is shown. **(C)** Migration of WT Eos suspended in medium alone or medium containing lactose or maltose toward murine eotaxin-1. Number of cells that migrated in each case was determined and expressed as the average number of cells/field. Combined data (mean ± SEM) from three independent experiments in duplicate is shown in **(A)** and **(C)**. ^#^*p* <0.05 versus WT Eos in **(A)**.

*In vitro* chemotaxis assays were performed to investigate whether Eos-expressed Gal-3 is essential for eotaxin-1-induced migration of Eos (**Figure 5A**). Relative to WT Eos, Gal-3^-/-^ Eos exhibited significantly decreased migration toward eotaxin-1 (*p* <0.05). Expression of CCR3, the eotaxin-1 receptor, by WT and Gal-3^-/-^ Eos was evaluated by flow cytometry and found to be similar in WT and Gal-3^-/-^ Eos (**Figure 5B**), indicating that decreased migration of Gal-3^-/-^ Eos is not due to reduced cell surface expression of CCR3. To confirm the involvement of intracellular Gal-3 in promoting eotaxin-1-induced migration, WT Eos were exposed to eotaxin-1 in the presence of lactose to block binding of any secreted Gal-3 to cell surface glycoproteins which could then induce migration. Migration of WT Eos in the presence of lactose was similar to that of untreated Eos or Eos treated with maltose (as control; **Figure 5C**). These studies suggest that intracellular Gal-3, rather than extracellular (secreted) Gal-3–glycan interactions, regulates Eos migration.

## DISCUSSION

Studies using mouse models have clearly demonstrated that endogenous Gal-3 plays a pro-inflammatory role during allergic asthma and chronic allergic airway inflammation (Zuberi et al., 2004; Ge et al., 2010). In these studies, relative to WT mice, development of allergen-induced airway eosinophilia, inflammation, AHR, and remodeling were all significantly attenuated in mice that were deficient in Gal-3. In the present study, we found that airway eosinophilia in acute and chronic allergen-challenged mice is accompanied with elevated levels of extracellular soluble Gal-3 in the BALF as well as cell associated and/or intracellular Gal-3 in the lung tissue. While Eos are the predominant inflammatory cells in the airways of allergen-challenged mice, inflammatory cells such as macrophages and T lymphocytes recruited to allergic airways are known to express and release Gal-3 (Liu et al., 1995; Joo et al., 2001). Our current studies suggest that mouse Eos also secrete Gal-3, especially when exposed to eotaxin-1, which together with the Gal-3 expressed and released by other inflammatory cells, accounts for the elevated levels of this molecule in the airway secretion during allergic inflammation.

Based on previous *in vivo* studies with Gal-3^-/-^ mice, Gal-3 appears to be required for recruitment of Eos to the airways in response to allergen challenge (Zuberi et al., 2004; Ge et al., 2010). While human Gal-3 is known to be a chemoattractant for macrophages (Sano et al., 2000), there is no evidence suggesting that Gal-3 can function directly as a chemoattractant for Eos. However, extracellularly, Gal-3 is known to bind to α4β1 on the cell surface of human Eos and function as an adhesion molecule to promote trafficking (rolling and adhesion) *in vitro* by interacting with VCAM-1 (Rao et al., 2007). Since Gal-3 is secreted by several cell types including Eos as shown here, secreted Gal-3 can bind to its glycan ligands on the Eos surface to mediate these events. We sought to determine the role of Eos-expressed Gal-3 (without the influence of secreted Gal-3 from other cells) specifically in regulating Eos trafficking and migration. BM-derived Eos from Gal-3^-/-^ mice rolled poorly on VCAM-1 relative to WT Eos under conditions of flow irrespective of similar level of expression of rolling receptors such as α4 and L-selectin in the two cell types indicating the requirement of Eos-expressed Gal-3 for efficient Eos rolling. Once localized on the cell surface, not only can Gal-3 interact directly with VCAM-1 (Tadokoro et al., 2009), but Gal-3 binding to α4 (Rao et al., 2007) may be necessary for efficient α 4–VCAM-1 interaction, thus resulting in poor cellular rolling when Gal-3 is absent. In addition, Gal-3^-/-^ Eos tended to adhere less efficiently to ICAM-1 than did WT Eos under conditions of flow, and consistent with this, expressed lower levels of integrin αM, the receptor that is known to mediate stable adhesion of Eos. Gal-3 has been shown to facilitate αM clustering on the cell surface (in lipid rafts) of Eos by cross-linking CD66b which is physically and constitutively associated with αM, thus promoting adhesion (Yoon et al., 2007). It is possible that in the absence of CD66b cross-linking by Gal-3, membrane localization of αM may be affected resulting in decreased adhesion. On the other hand, intracellular Gal-3 may regulate cell surface expression of αM. This is indeed the case for epidermal growth factor receptor (EGFR), where cytosolic Gal-3 was found to play a role in controlling intracellular trafficking and cell surface expression of this receptor to promote migration of keratinocytes. In the absence of Gal-3, the surface levels of EGFR are markedly reduced, with the receptor accumulating diffusely in the cytoplasm (Liu et al., 2012b).

In addition to poor rolling and decreased adhesion, Gal-3^-/-^ Eos adherent on VCAM-1 exhibited limited spreading and formation of membrane protrusions compared to WT Eos which may result in cell detachment under conditions of shear flow as evidenced by the reduced number of Gal-3^-/-^ Eos adherent on ICAM-1 in flow chamber assays. These differences in cell morphology of Gal-3^-/-^ Eos compared to WT Eos could be due to impaired cytoskeletal changes. Activation-induced changes in cell morphology enable spreading, stable cell adhesion and directed movement in response to a chemotactic gradient (Ridley et al., 2003; Rose et al., 2007; Huveneers and Danen, 2009). Consistent with the decreased spreading, the ability of Gal-3^-/-^ Eos to migrate in response to eotaxin-1 was significantly compromised. The level of expression of CCR3, however, was similar in Gal-3^-/-^ and WT Eos. Further, exposure of WT Eos to eotaxin-1 in the presence of lactose to prevent binding of any secreted Gal-3 did not alter migration suggesting that Eos migration is likely to be regulated by intracellular rather than extracellular Gal-3, thus accounting for the decreased migration of Gal-3^-/-^ Eos. A role for intracellular Gal-3 in regulating cell migration has also been demonstrated in various cancer cells by silencing Gal-3 expression (Kim et al., 2010; Wang et al., 2012; Zhang et al., 2013). Overall, signaling involving intracellular Gal-3 and/or secreted Gal-3 bound to the cell surface of Eos appears to be essential for Eos trafficking under flow and migration.

Not much is known regarding the role of intracellular Gal-3 in cell trafficking and migration. Kinases such as ERK2 and p38 are activated during eotaxin-1-induced Eos migration (Kampen et al., 2000) and in pancreatic cancer cells intracellular Gal-3 has been shown to activate Ras signaling, including down-stream phosphorylation of ERK, resulting in increased invasion (Song et al., 2012). It is possible that intracellular Gal-3 may play a role in Eos trafficking and migration by regulating specific kinases. In addition, recent studies indicate that intracellularly Gal-3 is phosphorylated in fibroblasts and phosphorylation of Gal-3 is not only required for localization at the cell periphery, an event important for cell migration (Hsu et al., 2009), but also for secretion of Gal-3 (Menon et al., 2011). Further, Gal-3 is a known substrate for c-Abl kinase in tumor cells (Balan et al., 2010). Taken together, it is possible that kinase activity is involved in the role played by Gal-3 in Eos trafficking and migration. However, detailed studies in Eos are needed to determine whether specific kinases regulate Gal-3 release or if Gal-3 is phosphorylated in activated Eos and if Gal-3 can potentially regulate other intracellular signaling molecules to affect Eos trafficking and migration. Finally, intracellular and extracellular Gal-3 is likely to have varied functions due to interaction with different ligands [interaction with glycans (extracellular) versus protein–protein interactions (intracellualar)]. For example, binding of secreted Gal-3 to its glycan ligands (e.g., integrins) on the cell surface can initiate signaling events such as activation of focal adhesion kinase (FAK) and phosphatidylinositol 3-kinase (PI3K) as well as increased F-actin turnover (Lagana et al., 2006). These are all important signaling events that occur during Eos trafficking (Huveneers and Danen, 2009; Kang et al., 2012) and could potentially be initiated by binding of Gal-3 to integrins such as α4β1.

In further support of our previous findings where allergen-challenged Gal-3^-/-^ mice have reduced airway eosinophilia, the current studies clearly establish a role for Eos-expressed Gal-3 in mediating Eos trafficking and migration *in vitro*.

## Conflict of Interest Statement

The authors declare that the research was conducted in the absence of any commercial or financial relationships that could be construed as a potential conflict of interest.
